# Different concentrations of fetal bovine serum affect cytokine modulation in Lipopolysaccharide-activated apical papilla cells in vitro^*^

**DOI:** 10.1590/1678-7757-2023-0020

**Published:** 2023-07-24

**Authors:** Letícia Martins SANTOS, Patricia e Silva CARDOSO, Elisa Abreu DINIZ, Juliana Garuba RAHHAL, Carla Renata SIPERT

**Affiliations:** 1 Universidade de São Paulo Departamento de Dentística Faculdade de Odontologia São Paulo SP Brasil Universidade de São Paulo, Departamento de Dentística, Faculdade de Odontologia, São Paulo, SP, Brasil.

**Keywords:** Cytokines, Fetal bovine serum, Apical papilla cells

## Abstract

Fetal bovine serum (FBS) is the most used supplement in culture media; however, it may interfere with in vitro assays via effects on cell proliferation and cytokine production. The ideal FBS concentration for assays using apical papilla cells (APCs) remains unknown. Therefore, this study aimed to evaluate the effects of FBS on APC activation, cell viability/proliferation, and cytokine production.

**Methodology:**

Human APCs were cultured, plated, and maintained in media containing increasing concentrations of FBS for 24 h, 48 h, 72 h, 7 days, and 14 days in the presence of Lipopolysaccharide (LPS - 1 µg/mL). At each time point, the cells were subjected to the MTT assay. The cytokines transforming growth factor (TGF)-β1, osteoprotegerin (OPG), and interleukin (IL)-6, along with the chemokine CCL2, were quantified using the enzyme-linked immunosorbent assay at the 24-h time-point. Statistical analysis was performed using two-way analysis of variance (ANOVA) followed by Tukey's post-hoc test (p<0.05).

**Results:**

In general, APCs exhibited increasing metabolic activity in an FBS concentration-dependent fashion, regardless of the presence of LPS. In contrast, FBS interfered with the production of all the cytokines evaluated in this study, affecting the response induced by the presence of LPS.

**Conclusion:**

FBS increased APC metabolism in a concentration-dependent manner and differentially affected the production of TGF-β1, OPG, IL-6, and CCL2 by APCs *in vitro*.

## Introduction

Apical papilla cells (APCs) have recently received increased attention in the field of Dentistry due to their proliferation, differentiation, and immunomodulatory potential. These cells have been studied with a particular interest in regenerative endodontics,^1,2^ and cytokine production has been a relevant research focus.^3,4^

Fetal bovine serum (FBS) is the most widely used medium supplement for cell culture methodologies mainly due to its high levels of growth-stimulating factors.^5^ However, FBS can interfere with phenomena such as cell proliferation and differentiation, modulation of molecules and cellular mechanisms, cell viability, and cytokine production.^6-11^ The mere presence of FBS, even without stimulation, was found able to constitutively induce the release of cytokines such as interleukin (IL)-6 and 8, usually expected in immunologically activated cells.^11^ These findings may reveal significant biases in cell culture methodologies; however, this behavior can vary significantly depending on cell population.

Cytokines have multiple biological properties.^12-14^ Transforming growth factor (TGF)-β1 represents a cytokine with anti-inflammatory properties.^15^ Osteoprotegerin (OPG) is known for its protective role in bone resorption.^16,17^ IL-6 is one of the most important pro-inflammatory cytokines,^18^ which release is induced by viral infections,^19^ trauma,^20^ and lipopolysaccharides (LPS).^21^ Monocyte chemotactic protein (CCL2/MCP-1) participates in monocyte chemotaxis and is, therefore, essential for osteoclast differentiation.

These four cytokines encompass different biological properties that are relevant to endodontic research, including anti-inflammatory and pro-inflammatory effects, along with impacts on bone metabolism. Another relevant aspect for the endodontics research community comes to the role of bacterial byproducts in APC behavior. LPS, a gram-negative bacteria byproduct, provides a rationale for *in vitro* cell activation, acting as an inducer.^22,23^ Therefore, this study aimed to investigate the effects of FBS used as a cell culture supplement on the viability, proliferation, and production of cytokines (such as TGF-β1, OPG, IL-6, and CCL2/MCP-1) in APC lipopolysaccharide-activated production *in vitro*.

## Methodology

### Cell culture

This study was approved by the local Ethics Committee (Process #4.251.087). Human APCs at passage six, previously characterized for their phenotype and function,^22^ were cultured in alpha-minimum essential medium (α-MEM) (Sigma-Aldrich, St Louis, MO) with 10% FBS (Gibco Life Technologies, Grand Island, NY) and antibiotics (100 µg/mL penicillin, 100 µg/mL streptomycin, 0.5 mg/mL amphotericin B – Invitrogen) under standard culture conditions (37°C, 100% humidity, 5% CO_2_, and 95% air).

### Cell characterization

Human APCs were phenotypically characterized by immunostaining for typical markers of mesenchymal stem cells (MSC) CD146, CD24, and STRO-1 (Cat No FAB932P; Cat No FAB5247A and MAB1038–SP followed by goat anti-mouse immunoglobulin M F0116, R&D Systems, Minneapolis, MN), and hematopoietic markers CD45 and CD34 (Cat No FAB1430P and Cat No FAB7227P, R&D Systems). Cells were analysed under flow cytometry (FACSCalibur; Becton Dickinson, San Jose, CA) and high expression of MSC-specific surface markers, along with low expression of the hematopoietic markers were detected.

APCs functionally characterization was confirmed by positive alizarin red S staining. Cells were plated in 24-well plates. The proliferation or differentiation medium was replaced for 14 days and, after this period, the mineralization analysis was performed using 40 mmol/L alizarin red S staining (CatA5533, Sigma-Aldrich) at a pH of 4.222.

### Cytotoxicity assay

The viability of APCs stimulated with different FBS concentrations was analyzed using the 3-(4,5-Dimethylthiazol-2-yl)-2,5-diphenyltetrazolium bromide (MTT) assay. APCs were seeded at 1.5×10^4^ cells per well in 48-well plates. After 24 h, the medium was replaced with 1% FBS α-MEM to allow cell adaptation. After another 24 h, the cells were stimulated with 1 µg/mL *Escherichia coli* LPS (Sigma-Aldrich, MO, USA)^23^ at increasing concentrations of FBS (0%, 0.5%, 1%, 10%, and 15%) in α-MEM in triplicate. After 24 h, 48 h, 72 h, 7 days, and 14 days of stimulation, the cell supernatant was replaced with 40 µL of an MTT solution (Sigma-Aldrich, St. Louis, MO, USA) (5 mg/mL) in phosphate-buffered saline (PBS) followed by 360 µL of 10% FBS α-MEM. The cells were incubated at 37°C for 4 h and protected from light. The MTT solution was discarded and replaced with 200 µL dimethyl sulfoxide (Synth, Diadema, SP, Brazil). Optical density was determined at a wavelength of 570 nm (Synergy HT, Biotek, Instruments, Inc. Winooski, VT, USA).

### Morphological analysis

APCs exposed to different FBS concentrations (0%, 0.5%, 1%, and 10%) were subjected to Fast Panoptic (Laborclin, SP, Brazil) staining technique morphological analysis. APCs were seeded at 1.5×10^4^ cells per well in 48-well plates and, after seven days of culture, they were fixed and stained following the manufacturer’s instructions. The center area of each well was selected, observed under light microscopy (Nikon TMS, Nikon, Tokyo, Japan), pictured (NIS Elements F, Nikon, Tokyo, Japan), and the cell density was qualitatively analyzed.

### Cytokine detection

APCs were seeded at a density of 5×10^4^ cells per well in 24-well plates with medium replacement, as described earlier. After 24 h, the cells were stimulated with LPS using 0%, 1%, or 10% FBS α-MEM.^23^ After 24 h, the cell supernatant was collected for quantitative analysis of TGF-β1, OPG, IL-6, and CCL2 using DuoSet enzyme-linked immunosorbent assay (ELISA) kits (R&D Systems), following the manufacturer’s instructions. For control purposes, cell-free medium was tested for cytokines. The data were normalized to 570 nm optical density. The experiment was conducted in quadruplicates.

### Statistical analysis

Statistical analyses were performed using GraphPad Prism 9.0 (GraphPad Software, San Diego, CA, USA). The Shapiro-Wilk test was used for normality testing, and the data were subjected to a two-way analysis of variance (ANOVA) followed by Tukey’s post-test. The significance level was set at p<0.05.

## Results

### Cell viability

**Figure 1:**
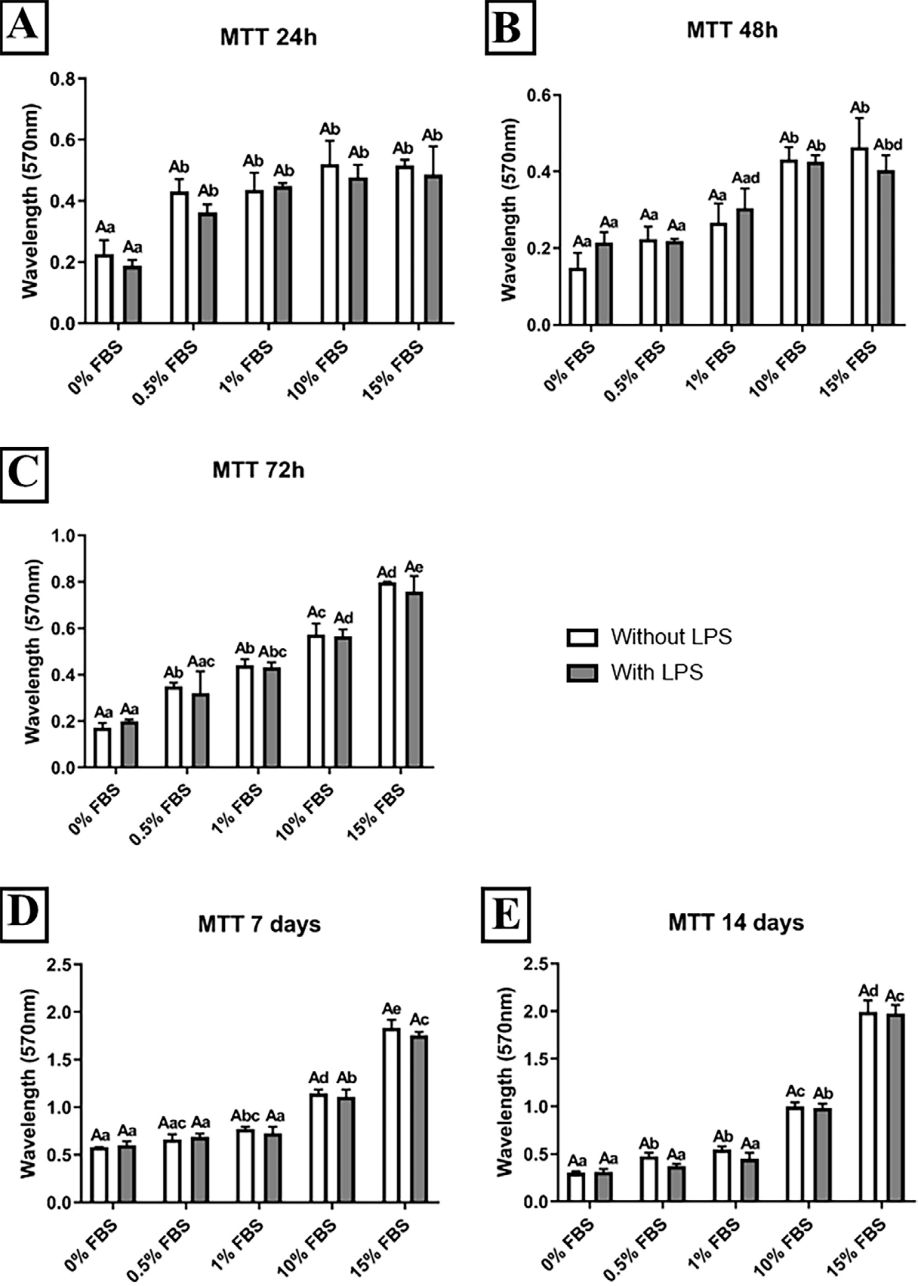
Viability / proliferation of Apical Papilla Cells activated with LPS. Absorbance (570 nm) data obtained from the MTT assay at experimental times of 24 h (A), 48 h (B), 72 h (C), 7 (D), and 14 days (E) in APC exposed to different concentrations of Fetal Bovine Serum (0; 0.5; 1; 10, and 15%) activated or not with LPS (1 µg/ml). The results showed the mean and standard deviation of the experiments performed in triplicate. Different capital letters represent statistical differences between groups with the same concentration of FBS. Different lowercase letters represent statistical differences between groups at different concentrations of FBS (Two-Way ANOVA with Tukey's test, p<0.05)

The results of the MTT assay are shown in Figure 1. LPS did not affect metabolic activity or viability regardless of FBS concentration and time point (Figure 1). At 24 h, APCs cultured with any tested concentration of FBS showed a significant improvement in metabolic activity compared to those cultured in FBS-free medium (Figure 1A). At 48 h, significant improvement was seen for 10% and 15% FBS media compared to the other conditions tested (Figure 1B). Considering the experimental periods of 72 h and greater, a gradual increase in metabolic activity was observed in a concentration-dependent manner for LPS-free samples (Figures 1C, D, and E). On the other hand, the same was not observed for LPS-activated cells (Figures 1C, D, and E). The interaction analysis for the FBS concentration and the presence of LPS was not significant.

### Morphological analysis

**Figure 2:**
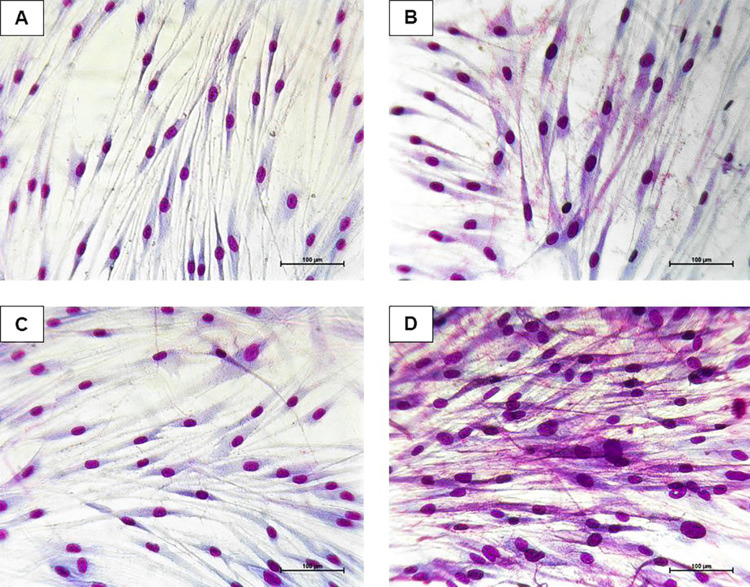
Apical Papilla Cells morphology. APC exposed to 0% FBS (A), 0,5% FBS (B), 1% FBS (C), and 10% FBS (D) for 7 days (Fast Panoptic staining. Calibration bar: 100 µm)

Although lower metabolic activity was observed in cells cultured without FBS (Figure 1), morphological analysis revealed the presence of nuclei and spindle-shaped cells that were similar to those in 0.5%, 1%, and 10% FBS samples, suggesting the maintenance of living APCs until 7 days of culture (Figure 2). Higher cellular densities were observed with increasing FBS concentrations, especially in the 10% FBS samples (Figure 2D).

### Cytokine production

**Figure 3:**
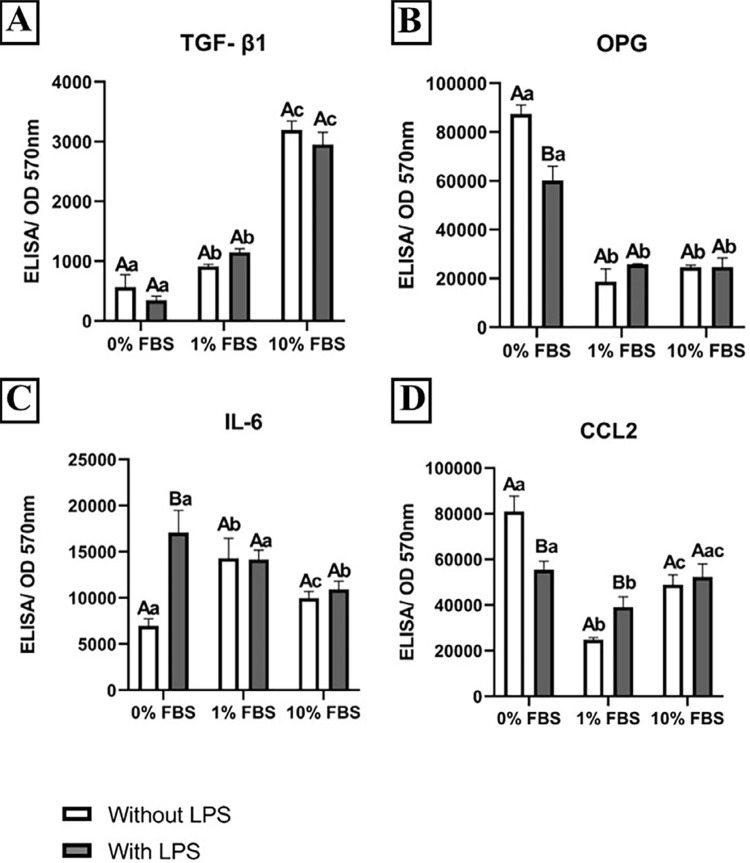
Cytokine production by LPS-activated Apical Papilla Cells. Mean and standard deviation of TGF-β1 (A), OPG (B), IL-6 (C), and CCL2 (D) concentrations according to the ELISA assay after 24h of exposure to different concentrations of Fetal Bovine Serum (0, 1, and 10%) in APC activated with LPS (1 µg/ml). Data normalized by the mean values of optical density (570 nm) obtained at MTT assay for the respective experimental condition. Experiments performed in sextuplicate. Different capital letters represent statistical differences between groups at the same FBS concentration. Different lowercase letters represent statistical differences between groups of different FBS concentrations (Two-Way ANOVA with Tukey's test, p<0.05)

**Figure 4:**
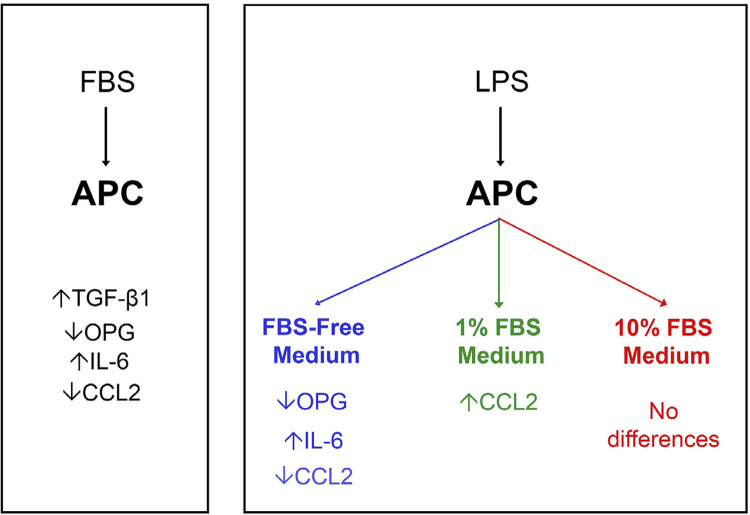
Fetal bovine serum effect on cytokines production by Apical Papilla Cells. The diagram summarizes the main findings. APC: apical papilla cells; FBS: Fetal Bovine Serum; TGF-β1: Transforming growth factor beta-1; OPG: Osteoprotegerin; IL-6: Interleukin 6; CCL2: chemokine CCL2; LPS: lipopolysaccharide

In general, medium supplementation with FBS resulted in an increase in TGF-β1 and IL-6 production by APCs (Figures 3A and 3C). However, we observed TGF-β1 production by APCs in an FBS concentration-dependent manner (Figure 3A), regardless of the presence of LPS. In contrast, when activated with LPS, APCs cultivated in serum-free medium decreased OPG (Figure 3B) and CCL2 release (Figure 3D) and increased the production of IL-6 (Figure 3C). Data from CCL2 and OPG production by APCs showed lower amounts of protein released by FBS-supplemented media (1% and 10%) (Figures 3B and 3D). It is important to highlight that the statistical differences observed for LPS-activated APCs for OPG, IL-6, and CCL2 were abrogated by the presence of FBS, even at a lower concentration (1%) (Figures 3B, C, and D). Interestingly, two-way ANOVA indicated a significant interaction between the presence of LPS and FBS content in the ELISA data (Figure 4).

## Discussion

APCs have been widely used in regenerative endodontic studies due to their proliferation, differentiation, and immunomodulatory potential.^1,2,24^ APCs are considered the key cell population involved in revascularization procedures and were first described in 2004.^25^ A better understanding of the behavior of such a cell population using *in vitro* studies is essential to achieve advances in clinical protocols and to improve the understanding of disease etiology.

Cell culture methodologies generally require medium supplementation with a serum of animal origin to provide viable conditions for proliferation, differentiation, and adhesion to plastic surfaces.^26^ FBS is a widely used supplement but it can interfere in *in vitro* experiments, affecting the quality, reproducibility, and reliability of the studies.^28^ Furthermore, nutrient deprivation is considered more critical than hypoxia for mesenchymal stem cell (MSC) survival.^10^ Our study showed that after a 24-h experimental time, APCs cultured in FBS-free medium exhibited lower metabolic activity than with any FBS concentration (Figure 1A). After 48 h and longer periods, a considerable increase in MTT reading for 10% and 15% FBS media was observed. In addition, optical density increased until 7 days of culture, even under serum-free conditions (Figure 1D). Taken together, results from the morphological analysis (Figure 2) suggest that cells were kept alive until day 7, and the optical density increase observed may be due to cell proliferation induced by FBS supplementation. Thus, for up to 7 days of culture, maintaining cells without FBS did not compromise cell viability even when activated with LPS. Our data corroborates with a recent study that demonstrated that cells grown in a 10% FBS supplemented medium proliferated more than those grown in serum-free media. The results showed that, despite decreased cell proliferation due to the absence of FBS, the cells were still able to maintain certain characteristics such as mineralization *in vitro*.^28^

The role of bacterial byproducts in APC behavior has recently received the attention of the endodontics research community. Reductions in the root canal microbiome can be achieved using enhanced antimicrobial protocols, but not the complete removal of bacterial remnants^29^ prior to bleeding induction into the pulp cavity.^29-31^ This provides a rationale for using LPS, a gram-negative bacteria byproduct, to induce *in vitro* cell activation^22,32^. We were concerned about the potential toxicity of LPS at the lower FBS concentrations. Thus, LPS was tested at viability assay varying FBS concentrations at different time intervals. Our results did not show metabolic alterations in activated APCs compared to the respective controls, regardless of the concentration of FBS. This finding is supported by the absence of interactions between the two variables analyzed in this trial (Figure 1). Therefore, any FBS concentration may be chosen for a future assay for cytokine detection. For this purpose, we chose concentrations of 1% and 10%, in addition to serum-free medium.

Regarding cytokine production, this study demonstrated that TGF-β1 release increased in a concentration-dependent manner based on FBS content (Figure 3A). Based on the absence of TGF-β1 in cell-free media (data not shown), our data suggest that the findings are not due to nonspecific reactions. TGF-β1 is a cytokine produced by several cell types with established roles in the regulation of other cytokines, including IL-6, OPG, and CCL2.^33-35^ Production of TGF-β1 by MSC is known to occur when stimulus, such as cytokines, products from viruses and bacteria, and derivatives of enzyme reactions, lead to activation of the Nuclear Factor-κB pathway.^36^ Recent literature has also demonstrated a higher production of TGF-β1 by MSCs when cultivated in a medium supplemented with amino acids.^37^ The presence of these molecules in the composition of FBS may be a major contributor to the mechanism underlying TGF-β1 induction in APCs by FBS. Interestingly, LPS did not affect TGF-β1 production in any of the FBS concentrations tested (Figure 3A).

OPG is a molecule that binds to RANKL (RANK ligand), a factor that promotes osteoclast formation, and prevents it from activating RANK [receptor activator of the (NF)-κB]. RANK is expressed on the cell surface of osteoclast precursors and its activation by RANKL induces osteoclast maturation. RANK/RANKL binding does not occur in the presence of OPG.^16,38^ Our data showed high levels of constitutive OPG, which, in turn, was downregulated by LPS only in FBS-free medium (Figure 3B). Interestingly, the addition of FBS downregulated OPG production (Figure 3B). At 1% FBS, we found upregulation of OPG production induced by LPS, whereas no difference was found at 10% FBS. Together with the significant interaction analysis rendered by two-way ANOVA, our data indicate that the amount of FBS in the culture medium hampers OPG production (Figure 3B), and thus may induce relevant methodological biases (Figure 4).

It is well known that IL-6 is one of the main pro-inflammatory cytokines produced by cells under infection or tissue damage.^14^ Accordingly, our data showed a significant increase in IL-6 production by LPS-activated APCs when compared to the respective LPS-free control. This activation effect was completely abrogated by FBS supplementation at both concentrations (Figure 3C). Additionally, FBS differentially affected IL-6 levels in both LPS-free and LPS-activated cells, depending on the serum concentration (Figure 4). As mentioned for OPG, the interaction analysis for IL-6 also demonstrated significant interference with FBS for LPS activation. Considering the composition of FBS; a variety of growth factors, cytokines, vitamins, hormones, and carbohydrates might be present, as well as endotoxins, infectious agents,^27,39^ and foreign proteins (e.g., xenogenic bovine serum antigens). Together, these factors may be responsible for inducing immune reactions in APCs (Figure 3C). Our findings also point to the relevant interference of FBS in IL-6 production by APCs *in vitro*.

CCL2 (monocyte chemoattractant protein-1, MCP-1) is a potent chemokine that activates signal transduction pathways, leading to monocyte chemotaxis.^40,41^ In contrast to the increase in IL-6 levels, constitutive CCL2 production by APCs was downregulated by LPS activation in FBS-free medium. As seen in the OPG data, this cytokine was present in lower levels in FBS-supplemented media than in FBS-free media. Additionally, the LPS activation response at 1% FBS was contrary to that observed in serum-free medium, and no response was observed for 10% FBS (Figure 4). Therefore, our CCL2 data also suggest that FBS interferes with APC chemokine production *in vitro* (Figure 4).

Serum-induced TGF-β1 production by APCs may underlie the modulation of immunological challenges due to its role in modulating other cytokines. Therefore, this effect might explain IL-6 inhibition,^35^ but not OPG and CCL2 reduction since TGF-β1 is described as being an OPG^33^ and CCL2^34^ inducer. Future studies should be conducted on other components and mechanisms involved in the modulation of cytokine release by FBS.

Taken together, our findings demonstrate that FBS supplementation may interfere with cell proliferation and cytokine production *in vitro*. By modulating TGF-β1, IL-6, OPG, and CCL2 release, FBS may create a microenvironment *in vitro* that differs from *in vivo* conditions (Figure 4). Therefore, we speculate that immunological activation in this context may be significantly underestimated. Studies that aim to investigate the immunological activation of cells *in vitro* should consider avoiding it or at least first test FBS concentrations to reduce biases when using similar methodologies. Even considering the outstanding efforts of researchers in developing substitutes, FBS remains the most commonly used supplement for cell culture.^30,42^ The final decision regarding FBS usage and concentration should be made according to the target cell and molecules of each study.

## Conclusion

In summary, this study showed that FBS supplementation may interfere with APC proliferation and cytokine production *in vitro*.
